# Supplementary figures and images for: Identification of a novel inhibitor of SARS-CoV-2 3CL-PRO through virtual screening and molecular dynamics simulation
Source: PeerJ. 2021 Apr 13;9:e11261. doi: 10.7717/peerj.11261 (PMC8051358; doi:10.7717/peerj.11261)

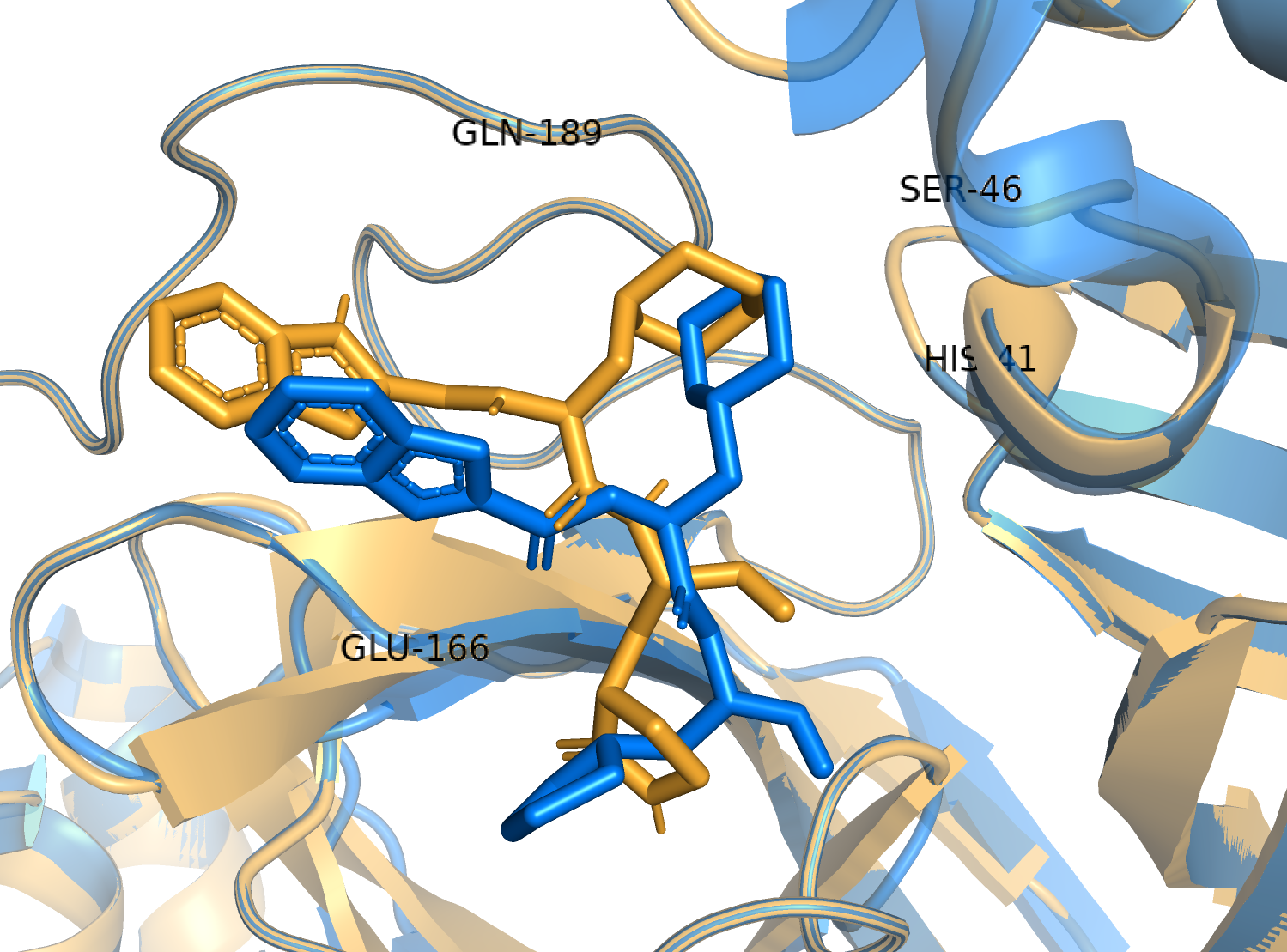

Supplement: Supplemental Information 1 [file peerj-09-11261-s001.png]

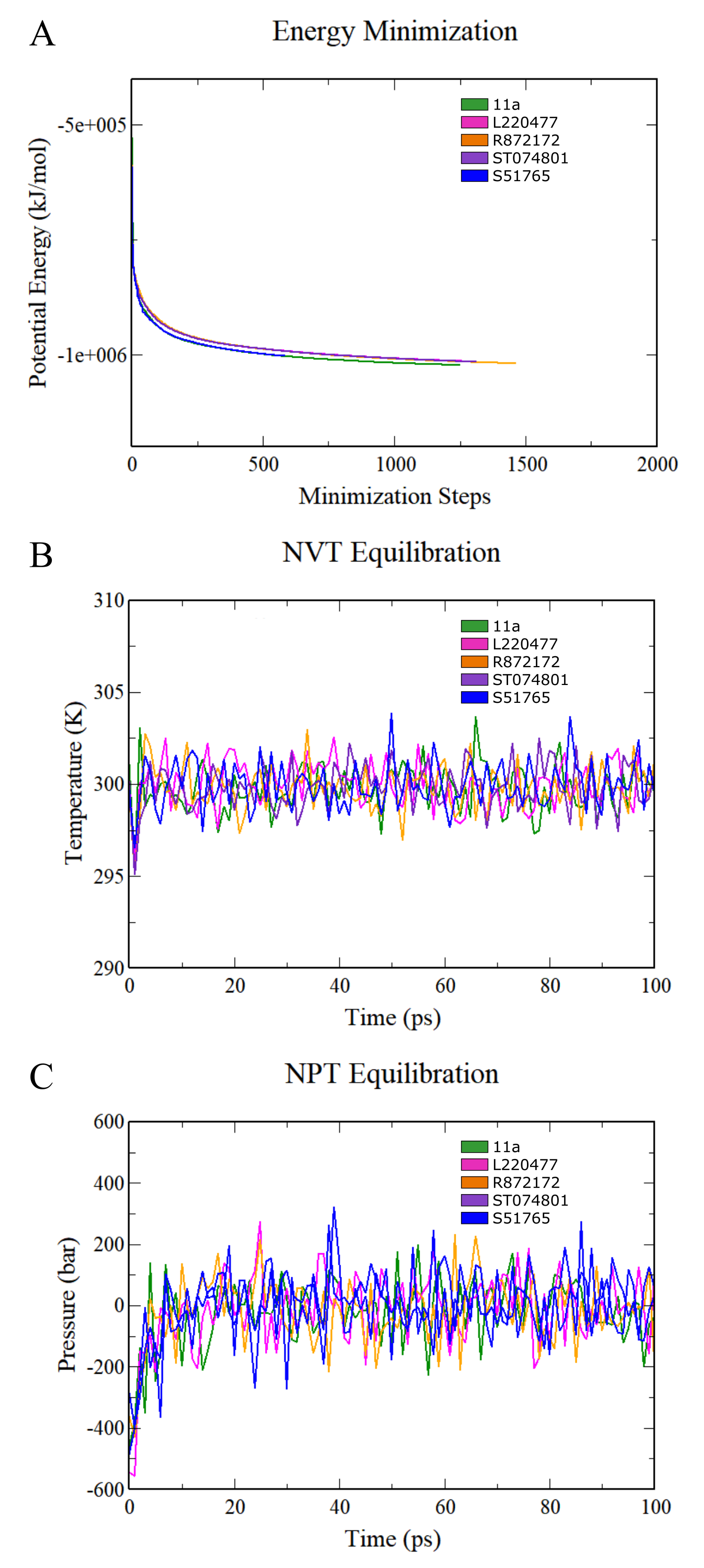

Supplement: Supplemental Information 2 — (A) Energy minimization. (B) NVT equilibration. (C) NPT equilibration. [file peerj-09-11261-s002.png]

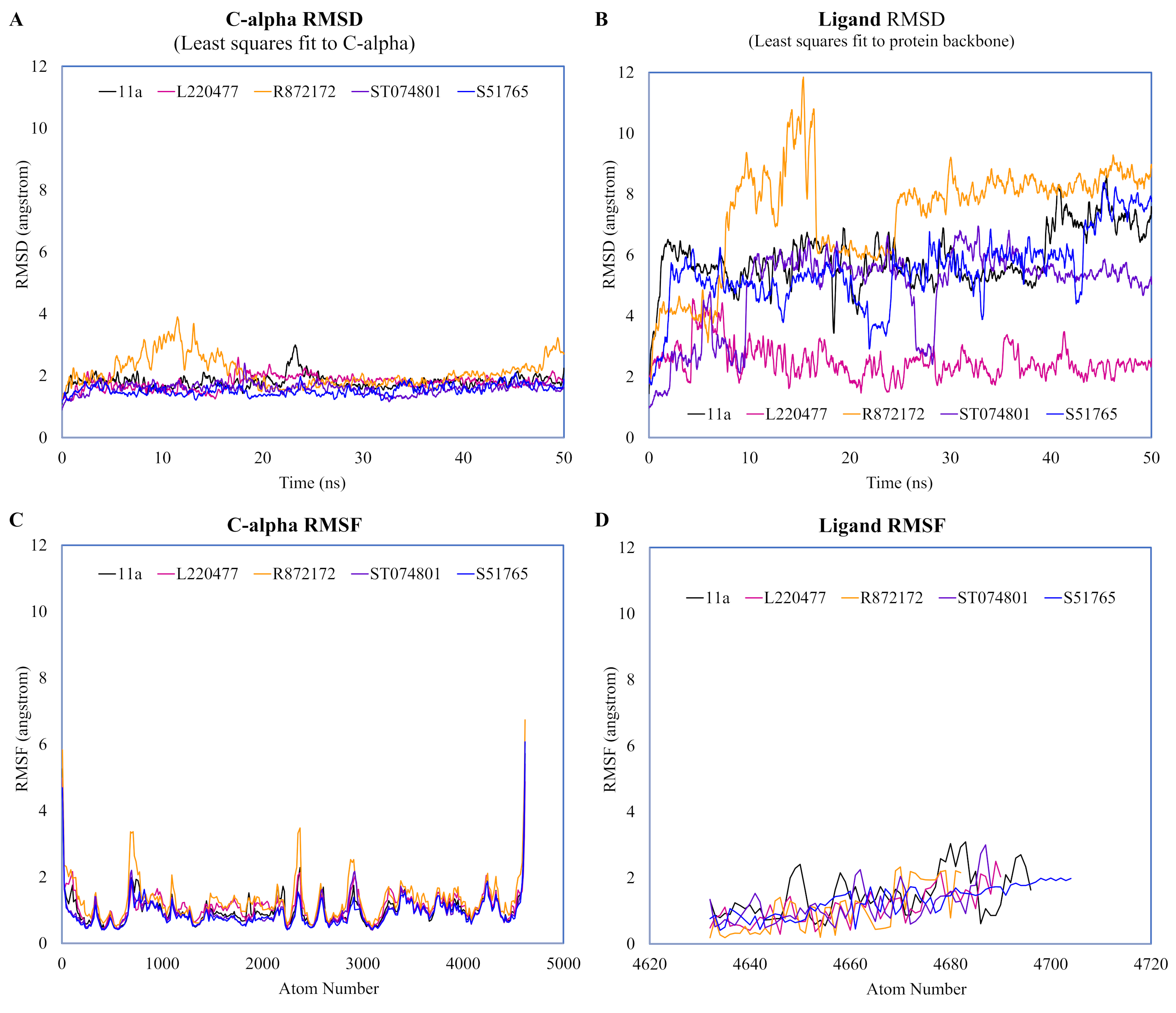

Supplement: Supplemental Information 3 [file peerj-09-11261-s003.png]

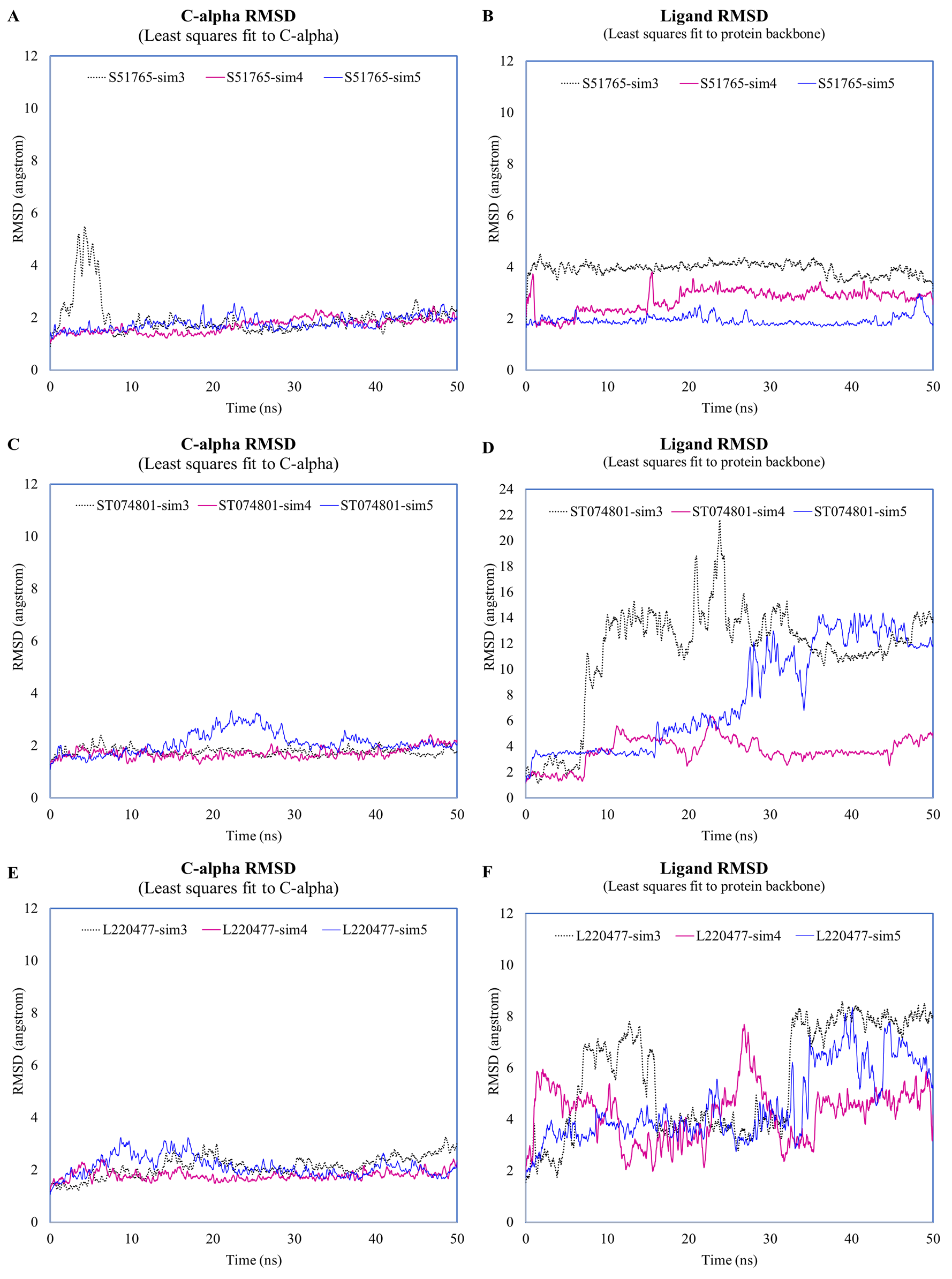

Supplement: Supplemental Information 4 — c-alpha RMSD and ligand RMSD for S51765 (A, B), ST074801 (C, D), and L220477 (E, F). [file peerj-09-11261-s004.png]

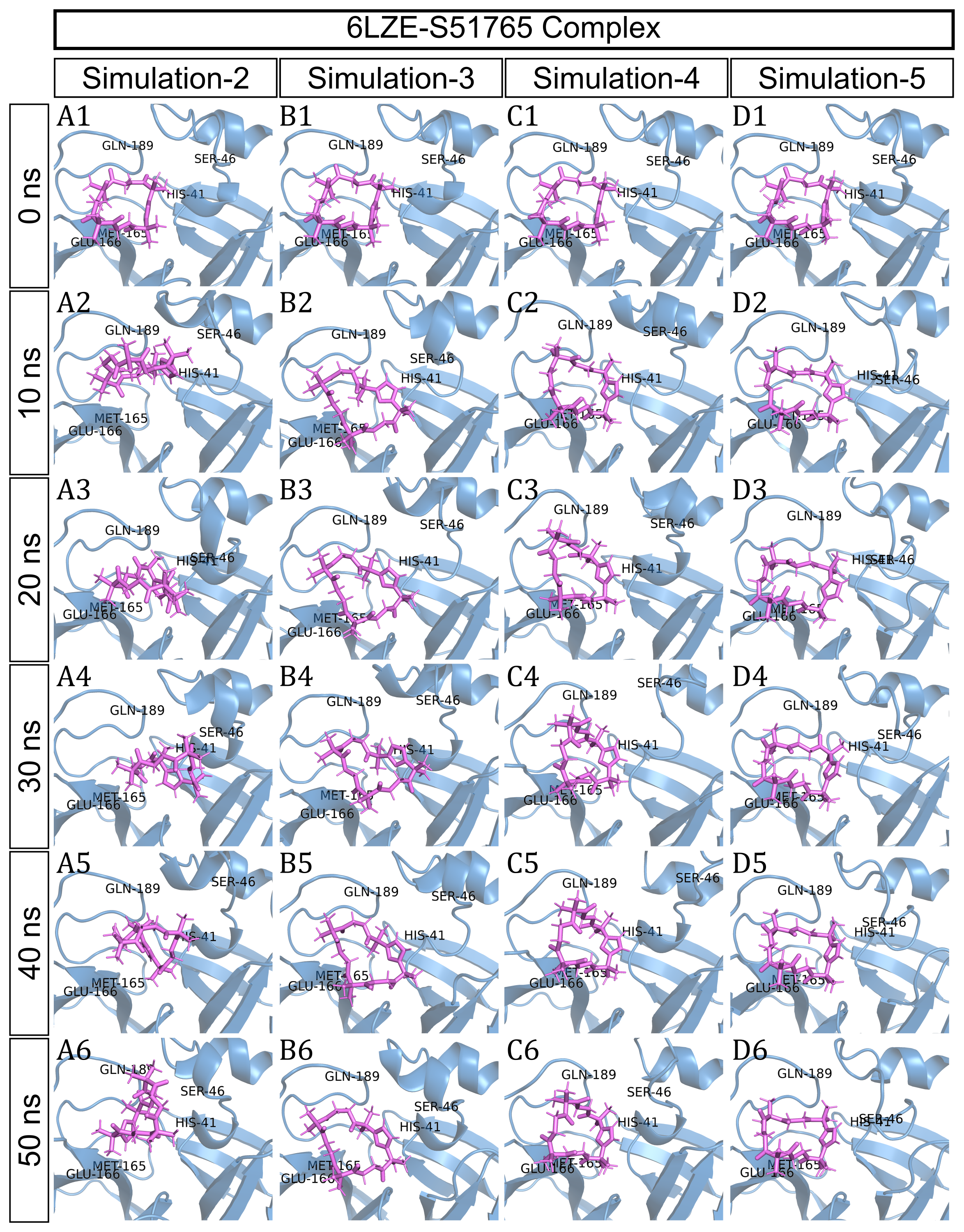

Supplement: Supplemental Information 5 [file peerj-09-11261-s005.png]

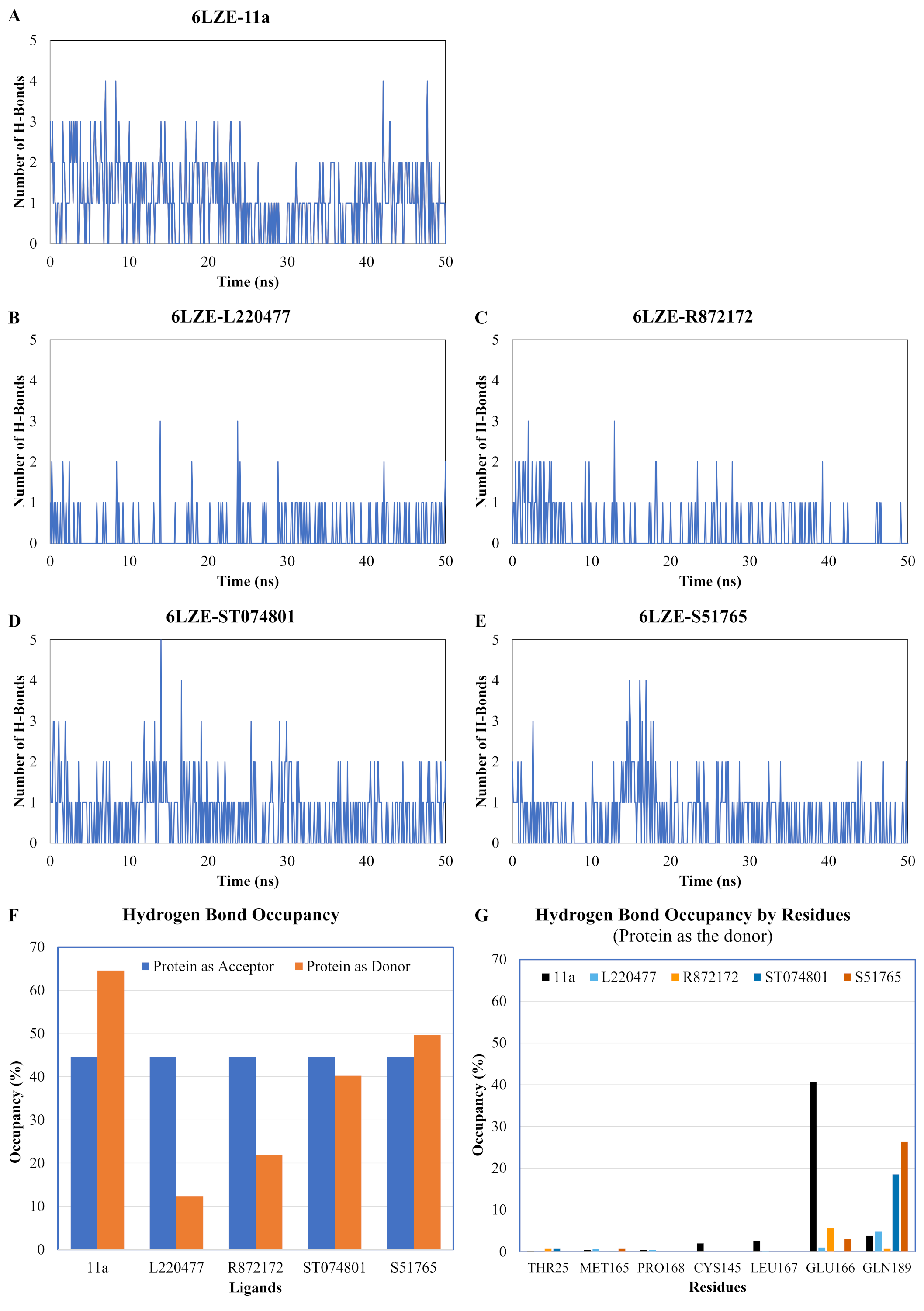

Supplement: Supplemental Information 6 [file peerj-09-11261-s006.png]

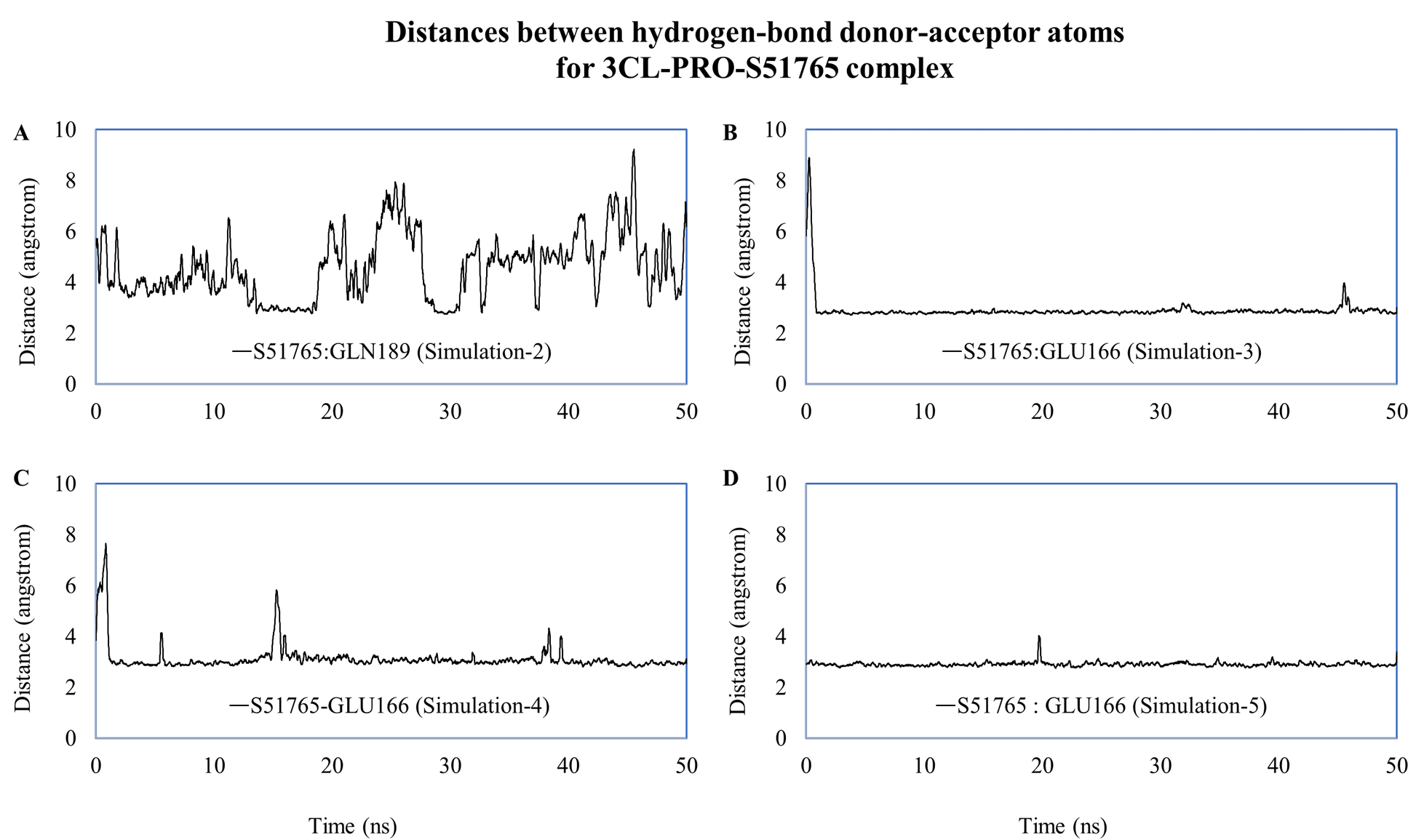

Supplement: Supplemental Information 7 [file peerj-09-11261-s007.png]
