# Supplementary material for: Identification of a novel inhibitor of SARS-CoV-2 3CL-PRO through virtual screening and molecular dynamics simulation
Source: PeerJ. 2021 Apr 13;9:e11261. doi: 10.7717/peerj.11261 (PMC8051358; doi:10.7717/peerj.11261)
Supplement: Supplementary file 2 [file peerj-09-11261-s008.odt]

|  |  | Predicted Binding Affinity (kcal/mol) |
| --- | --- | --- |
| Group | Molecule | Total | Single Crystal Structures | MD Simulation Structures (Average of ten structures) |
| Average | SD | 6LZE | 6M0K | 6YB7 | 6LZE_MD | 6M0K_MD | 6BY7_MD |
| Vina Hits | R897698 | -8.6 | 0.5 | -9.6 | -8.6 | -8.2 | -8.92 | -8.15 | -8.78 |
| Vina Hits | ST031238 | -8.4 | 0.5 | -9.2 | -9 | -8.3 | -8.56 | -8.12 | -8.37 |
| Vina Hits | ST018363 | -8.2 | 0.7 | -9.7 | -8.8 | -8.5 | -8.59 | -7.59 | -8.25 |
| Vina Hits | R818984 | -8.2 | 0.6 | -9.1 | -8.7 | -8.8 | -8.39 | -7.72 | -8.22 |
| Vina Hits | ST088323 | -8.1 | 0.5 | -8.7 | -8.4 | -8.5 | -8.28 | -7.6 | -8.26 |
| Vina Hits | L363340 | -8.1 | 0.6 | -9.2 | -8.4 | -7.8 | -8.18 | -7.6 | -8.3 |
| Vina Hits | ST042014 | -8.1 | 0.5 | -8.5 | -8.1 | -9.2 | -8.2 | -7.83 | -7.98 |
| Vina Hits | ST031351 | -8.0 | 0.5 | -8.1 | -7.9 | -9.2 | -8.25 | -7.69 | -8.08 |
| Vina Hits | L220477 | -8.0 | 0.8 | -9.4 | -8.7 | -7.8 | -8.3 | -7.24 | -8.28 |
| Vina Hits | R679445 | -8.0 | 0.6 | -8.6 | -8.1 | -8.3 | -8.18 | -7.49 | -8.22 |
| Vina Hits | ST000954 | -8.0 | 0.6 | -9 | -8.8 | -8.8 | -8.24 | -7.39 | -8.09 |
| Vina Hits | ST020475 | -8.0 | 0.6 | -9.3 | -8.9 | -8.1 | -8.17 | -7.42 | -8.12 |
| Vina Hits | ST018407 | -8.0 | 0.5 | -9.1 | -8.4 | -8.5 | -7.98 | -7.71 | -8.04 |
| Vina Hits | L128643 | -8.0 | 0.6 | -9.9 | -8.4 | -8.4 | -8.02 | -7.62 | -7.95 |
| Vina Hits | ST094780 | -7.9 | 0.6 | -8.6 | -8.2 | -8.4 | -8.26 | -7.31 | -8.1 |
| Vina Hits | ST074801 | -7.9 | 0.6 | -8.6 | -8.4 | -8.5 | -8.01 | -7.41 | -8.18 |
| Vina Hits | R461083 | -7.9 | 0.5 | -9 | -8.4 | -8.3 | -8.11 | -7.51 | -7.95 |
| Vina Hits | R872172 | -7.8 | 0.6 | -8.5 | -8.8 | -8.4 | -7.93 | -7.27 | -8.01 |
| Vina Hits | ST074799 | -7.8 | 0.6 | -9 | -8.1 | -8.6 | -7.93 | -7.17 | -8.07 |
| Vina Hits | S51765 | -7.7 | 0.7 | -9.1 | -8.2 | -6.8 | -8.1 | -7.08 | -7.8 |
| Decoy | C15498341 | -7.6 | 0.5 | -8.6 | -7.3 | -7.6 | -7.7 | -7.28 | -7.64 |
| Decoy | C20570172 | -7.5 | 0.7 | -8.3 | -7.3 | -7.7 | -7.62 | -6.79 | -7.91 |
| Decoy | C02520392 | -7.5 | 0.5 | -8.6 | -7.4 | -7.5 | -7.55 | -7.27 | -7.48 |
| Decoy | C16293465 | -7.4 | 0.6 | -8.1 | -6.6 | -7.7 | -7.59 | -7.06 | -7.45 |
| Decoy | C03797930 | -7.3 | 0.7 | -8.2 | -8.7 | -7.2 | -7.42 | -6.64 | -7.7 |
| Decoy | C83318194 | -7.3 | 0.4 | -8.1 | -7.6 | -7.8 | -7.53 | -6.74 | -7.38 |
| Decoy | C09198485 | -7.3 | 0.6 | -8.3 | -7.1 | -7.8 | -7.48 | -6.66 | -7.51 |
| Decoy | C12502781 | -7.3 | 0.5 | -7.6 | -7.3 | -7.4 | -7.45 | -6.95 | -7.33 |
| Decoy | C08873546 | -7.2 | 0.4 | -8.1 | -7.4 | -7.8 | -7.3 | -6.96 | -7.27 |
| Reference Active | 11a_ref_active | -7.2 | 0.6 | -7.8 | -7.9 | -7.1 | -7.56 | -6.64 | -7.38 |
| Decoy | C16320893 | -7.1 | 0.5 | -7.9 | -7.7 | -7.2 | -7.33 | -6.81 | -7.16 |
| Decoy | C09240148 | -7.1 | 0.4 | -8 | -7.3 | -7.3 | -7.21 | -6.94 | -7.11 |
| Decoy | C09891882 | -7.1 | 0.5 | -8.3 | -7.6 | -7.6 | -7.21 | -6.75 | -7.19 |
| Decoy | C16632549 | -7.1 | 0.6 | -8.2 | -7.2 | -6.7 | -7.14 | -6.64 | -7.36 |
| Decoy | C71901589 | -7.0 | 0.5 | -7.8 | -7.1 | -7.8 | -7.27 | -6.58 | -7.05 |
| Decoy | C09620230 | -7.0 | 0.5 | -7.7 | -7.1 | -7.5 | -7.34 | -6.71 | -6.87 |
| Decoy | C03846613 | -7.0 | 0.6 | -7.7 | -7.1 | -7.3 | -7.27 | -6.3 | -7.21 |
| Decoy | C22570349 | -7.0 | 0.5 | -7.9 | -6.8 | -6.6 | -7.17 | -6.59 | -7.08 |
| Decoy | C40683522 | -6.9 | 0.5 | -7.6 | -7 | -6.7 | -7.14 | -6.65 | -7.01 |
| Decoy | C09026939 | -6.9 | 0.4 | -7.6 | -7 | -7.4 | -7.13 | -6.55 | -6.99 |
| Decoy | C38881187 | -6.9 | 0.5 | -8.1 | -6.9 | -7.1 | -6.88 | -6.75 | -7.02 |
| Decoy | C33871869 | -6.9 | 0.5 | -7.5 | -7.1 | -7.1 | -6.94 | -6.54 | -7.17 |
| Decoy | C20528314 | -6.9 | 0.7 | -8.2 | -6.7 | -7.2 | -6.93 | -6.43 | -7.18 |
| Decoy | C58133586 | -6.9 | 0.6 | -8 | -7.3 | -7.4 | -7.08 | -6.56 | -6.83 |
| Decoy | C21316414 | -6.8 | 0.6 | -7.9 | -6.9 | -7.1 | -6.93 | -6.4 | -7.07 |
| Decoy | C09409893 | -6.8 | 0.4 | -7.3 | -6.6 | -7.1 | -6.87 | -6.62 | -6.96 |
| Decoy | C21323121 | -6.8 | 0.7 | -7.3 | -7 | -7.4 | -6.81 | -6.41 | -7.16 |
| Decoy | C20528155 | -6.8 | 0.5 | -7.4 | -6.5 | -6.5 | -6.98 | -6.54 | -6.8 |
| Decoy | C13121226 | -6.8 | 0.6 | -7.4 | -6.7 | -7 | -6.81 | -6.38 | -7.01 |
| Decoy | C58998229 | -6.7 | 0.5 | -7.7 | -6.8 | -6.3 | -6.86 | -6.58 | -6.75 |
| Decoy | C58997867 | -6.7 | 0.6 | -7.7 | -7.3 | -7.3 | -6.77 | -6.53 | -6.7 |
| Decoy | C03524549 | -6.7 | 0.7 | -7.9 | -7.6 | -6.6 | -7.07 | -6.18 | -6.67 |
| Decoy | C01111485 | -6.7 | 0.5 | -7.5 | -7.2 | -6.6 | -6.84 | -6.05 | -7.08 |
| Decoy | C58998128 | -6.7 | 0.4 | -7.1 | -6.6 | -6.5 | -6.82 | -6.44 | -6.77 |
| Decoy | C20527302 | -6.6 | 0.6 | -7.3 | -6.2 | -6.4 | -6.61 | -6.45 | -6.88 |
| Decoy | C59312095 | -6.6 | 0.5 | -7.6 | -6.6 | -6.3 | -6.63 | -6.46 | -6.68 |
| Decoy | C04388726 | -6.6 | 0.5 | -7.2 | -6.9 | -7.4 | -6.73 | -6.04 | -6.86 |
| Decoy | C40155905 | -6.6 | 0.6 | -8 | -7.3 | -7.6 | -6.74 | -5.93 | -6.71 |
| Decoy | C16811033 | -6.5 | 0.5 | -7.1 | -6.9 | -6.9 | -6.79 | -6.03 | -6.62 |
| Decoy | C09128471 | -6.5 | 0.8 | -7.2 | -7.1 | -7.1 | -6.79 | -6 | -6.59 |
| Decoy | C58988384 | -6.4 | 0.6 | -7.1 | -6 | -6.4 | -6.64 | -5.99 | -6.49 |
| Decoy | C25073536 | -6.4 | 0.4 | -7 | -6.4 | -6.8 | -6.38 | -6.14 | -6.46 |
| Decoy | C20292952 | -6.3 | 0.7 | -7.8 | -6.1 | -6.5 | -6.36 | -5.93 | -6.6 |
| Decoy | C13763634 | -6.3 | 0.5 | -7.4 | -6.7 | -6.9 | -6.6 | -5.83 | -6.38 |
| Decoy | C20917273 | -6.2 | 0.8 | -7.7 | -5.6 | -6.6 | -6.26 | -6.08 | -6.12 |
| Decoy | C58989519 | -6.2 | 0.7 | -6.7 | -7.2 | -6.2 | -6.19 | -5.83 | -6.3 |
| Decoy | C12495322 | -6.2 | 0.5 | -6.7 | -6.1 | -6 | -6.37 | -5.76 | -6.32 |
| Decoy | C20917866 | -6.1 | 0.6 | -6.7 | -5.8 | -6.3 | -6 | -5.79 | -6.57 |
| Decoy | C09820856 | -6.1 | 0.4 | -6.4 | -6.3 | -5.7 | -6.32 | -5.84 | -6.19 |
| Decoy | C14236347 | -6.1 | 0.5 | -7.1 | -7 | -7 | -6.21 | -5.68 | -6.17 |
| Decoy | C05638191 | -5.7 | 0.4 | -6.8 | -6.1 | -6.3 | -5.7 | -5.26 | -5.88 |
