# Supplementary material for: Identification of a novel inhibitor of SARS-CoV-2 3CL-PRO through virtual screening and molecular dynamics simulation
Source: PeerJ. 2021 Apr 13;9:e11261. doi: 10.7717/peerj.11261 (PMC8051358; doi:10.7717/peerj.11261)
Supplement: Supplementary file 3 [file peerj-09-11261-s009.odt]

| Acceptor | Donor | Hydrogen Bonding Occupancy (%) |
| --- | --- | --- |
| 11a | L220477 | R872172 | ST074801 | S51765 |
| ARG188-Main | Ligand | 0.2 | 0.6 | 0.2 | 5.19 | 0.6 |
| ASN119-Side | Ligand |  |  | 1.4 |  |  |
| ASN142-Side | Ligand | 0.2 |  |  |  | 0.4 |
| ASN51-Main | Ligand |  | 0.4 |  |  |  |
| ASP187-Main | Ligand | 0.2 | 10.78 | 0.2 | 2.2 |  |
| ASP48-Main | Ligand |  |  | 0.2 |  |  |
| CYS145-Side | Ligand | 0.2 |  |  |  |  |
| CYS44-Main | Ligand | 12.38 |  | 0.2 |  |  |
| GLN189-Main | Ligand |  | 0.8 |  | 1 |  |
| GLN189-Side | Ligand | 2.99 |  |  | 0.2 | 0.8 |
| GLN192-Side | Ligand |  |  |  | 0.4 |  |
| GLU166-Main | Ligand | 1.2 | 0.6 |  | 0.2 | 6.59 |
| GLU166-Side | Ligand | 0.8 |  |  |  |  |
| HIS164-Main | Ligand | 3.19 |  |  |  |  |
| HIS164-Side | Ligand |  |  |  | 0.2 |  |
| HIS41-Main | Ligand | 0.2 |  |  |  | 0.2 |
| HIS41-Side | Ligand | 0.4 |  |  |  | 1.8 |
| LEU167-Side | Ligand |  |  |  | 0.2 |  |
| MET49-Main | Ligand |  | 0.4 | 0.4 |  |  |
| PHE140-Main | Ligand | 0.2 |  | 0.2 |  |  |
| SER144-Side | Ligand | 0.4 |  |  |  |  |
| SER46-Main | Ligand | 0.2 |  | 0.2 |  |  |
| SER46-Side | Ligand | 1.4 |  | 0.2 |  |  |
| THR190-Main | Ligand |  | 0.2 |  | 2.2 |  |
| THR24-Side | Ligand |  |  | 0.4 |  |  |
| THR25-Side | Ligand | 3.39 |  | 1 |  |  |
| THR26-Main | Ligand |  | 0.6 |  |  |  |
| THR45-Main | Ligand | 1.2 | 0.2 |  | 1 |  |
| TYR54-Side | Ligand |  | 0.8 |  |  |  |
| VAL186-Main | Ligand | 0.4 |  |  |  |  |
| Ligand | ALA191-Main |  | 1.39 |  | 0.6 |  |
| Ligand | ARG188-Main |  |  | 0.2 | 0.2 |  |
| Ligand | ASN119-Side |  |  | 1.2 |  |  |
| Ligand | ASN142-Main |  |  | 0.4 |  |  |
| Ligand | ASN142-Side | 3.19 |  | 2.19 |  |  |
| Ligand | ASN51-Main |  | 0.2 |  |  |  |
| Ligand | CYS145-Main | 2.19 |  |  |  |  |
| Ligand | CYS145-Side | 1.79 |  |  |  | 1.99 |
| Ligand | CYS44-Side |  | 0.6 | 0.6 |  | 0.6 |
| Ligand | GLN189-Main |  | 0.8 |  | 7.37 | 1 |
| Ligand | GLN189-Side | 0.6 | 4.98 | 2.99 | 11.16 | 1.99 |
| Ligand | GLN192-Main | 0.4 |  |  | 8.37 |  |
| Ligand | GLN192-Side |  | 0.6 |  | 6.57 | 0.4 |
| Ligand | GLU166-Main | 16.14 | 0.4 | 0.4 | 0.2 | 57.17 |
| Ligand | GLU166-Side |  |  | 0.2 |  | 0.2 |
| Ligand | GLY143-Main | 0.4 |  |  |  |  |
| Ligand | HIS164-Side |  |  |  | 0.2 |  |
| Ligand | HIS172-Side | 0.4 |  |  |  |  |
| Ligand | HIS41-Side | 19.12 | 1.99 |  |  | 0.2 |
| Ligand | LEU167-Side |  | 0.6 |  |  |  |
| Ligand | LEU27-Side | 0.4 |  | 0.2 |  |  |
| Ligand | MET165-Main | 0.8 |  |  |  |  |
| Ligand | MET165-Side | 0.2 | 0.4 |  |  | 2.39 |
| Ligand | MET49-Main |  |  |  | 0.6 |  |
| Ligand | MET49-Side | 0.2 |  |  | 0.4 | 0.6 |
| Ligand | PHE140-Side | 0.2 |  |  |  |  |
| Ligand | PRO168-Side |  | 1.79 |  |  |  |
| Ligand | SER46-Main | 0.2 |  | 0.4 | 0.6 |  |
| Ligand | SER46-Side | 0.8 | 0.2 |  | 1.2 | 0.2 |
| Ligand | THR190-Main |  | 3.19 |  | 5.58 |  |
| Ligand | THR190-Side |  | 0.4 |  |  |  |
| Ligand | THR24-Side |  |  | 5.38 |  |  |
| Ligand | THR25-Main |  |  | 0.2 |  |  |
| Ligand | THR25-Side | 1.39 |  | 0.2 | 0.4 |  |
| Ligand | THR26-Main |  |  | 1.39 |  |  |
| Ligand | THR26-Side |  |  | 0.2 |  |  |
| Ligand | TYR54-Side |  |  | 0.4 |  |  |
| Total | 77.57 | 32.92 | 21.15 | 56.24 | 77.13 |
