# Supplementary material for: Identification of a novel inhibitor of SARS-CoV-2 3CL-PRO through virtual screening and molecular dynamics simulation
Source: PeerJ. 2021 Apr 13;9:e11261. doi: 10.7717/peerj.11261 (PMC8051358; doi:10.7717/peerj.11261)
Supplement: Supplementary file 4 [file peerj-09-11261-s010.odt]

| Rank | Ligand_Name | Predicted Binding Affinity (kcal/mol) | SD (±) | 6LZE_Rank | 6M0K_Rank | 6YB7_Rank |
| --- | --- | --- | --- | --- | --- | --- |
| 1 | R897698 | -8.7 | 0.5 | 3 | 5 | 63 |
| 2 | ST031238 | -8.4 | 0.5 | 17 | 12 | 87 |
| 3 | ST042014 | -8.4 | 0.6 | 2 | 1 | 85 |
| 4 | ST018363 | -8.3 | 0.7 | 4 | 9 | 28 |
| 5 | L363340 | -8.2 | 0.6 | 18 | 35 | 178 |
| 6 | ST031351 | -8.1 | 0.5 | 107 | 179 | 3 |
| 7 | L220477 | -8.1 | 0.8 | 8 | 11 | 142 |
| 8 | R679445 | -8.1 | 0.6 | 80 | 24 | 29 |
| 9 | ST000954 | -8.1 | 0.6 | 42 | 37 | 13 |
| 10 | R872172 | -8.1 | 0.6 | 19 | 6 | 41 |
| 11 | ST074801 | -8.1 | 0.5 | 56 | 143 | 126 |
| 12 | ST088323 | -8.1 | 0.5 | 57 | 15 | 43 |
| 13 | ST018407 | -8.1 | 0.6 | 81 | 13 | 12 |
| 14 | S51765 | -8.0 | 0.6 | 135 | 88 | 22 |
| 15 | R818984 | -8.0 | 0.7 | 20 | 4 | 5 |
| 16 | ST074799 | -8.0 | 0.5 | 82 | 112 | 8 |
| 17 | ST094780 | -8.0 | 0.6 | 136 | 89 | 68 |
| 18 | ST020475 | -8.0 | 0.6 | 12 | 3 | 62 |
| 19 | L128643 | -8.0 | 0.7 | 1 | 32 | 30 |
| 20 | R461083 | -8.0 | 0.5 | 28 | 36 | 64 |
